# A High‐Yielding Synthesis of EIDD‐2801 from Uridine**

**DOI:** 10.1002/ejoc.202001340

**Published:** 2020-11-12

**Authors:** Alexander Steiner, Desiree Znidar, Sándor B. Ötvös, David R. Snead, Doris Dallinger, C. Oliver Kappe

**Affiliations:** ^1^ Institute of Chemistry University of Graz, NAWI Graz Heinrichstrasse 28 8010 Graz Austria; ^2^ Center for Continuous Flow Synthesis and Processing (CCFLOW) Research Center Pharmaceutical Engineering GmbH (RCPE) Inffeldgasse 13 8010 Graz Austria; ^3^ Medicines for All Institute 737 N. 5 23298‐0100 Richmond Virginia USA

**Keywords:** EIDD‐2801, COVID‐19, Continuous flow, Acetonide deprotection, Triazolation

## Abstract

A simple reordering of the reaction sequence allowed the improved synthesis of EIDD‐2801, an antiviral drug with promising activity against the SARS‐CoV‐2 virus, starting from uridine. Compared to the original route, the yield was enhanced from 17 % to 61 %, and fewer isolation/purification steps were needed. In addition, a continuous flow procedure for the final acetonide deprotection was developed, which proved to be favorable toward selectivity and reproducibility.

## Graphical abstract

An improved protocol was developed for the synthesis of EIDD‐2801, which is currently in Phase II clinical trials for the treatment of COVID‐19. The process employs uridine as starting material and proceeds through triazolation, one‐pot acetonide protection/esterification and telescoped hydroxyamination/continuous flow acetonide deprotection.

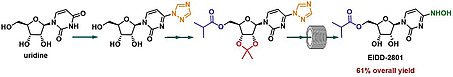

The current medical crisis caused by the SARS‐CoV‐2 virus, has impacted people's lives and economy worldwide, with presently more than 35 million confirmed cases.^[^ ^1^ ^]^ In view of the severity of the COVID‐19 pandemic, there is a continuous quest for new drugs for the treatment of this disease. EIDD‐2801, a NHC (β‐d‐N^4^‐hydroxycytidine) prodrug, has shown potent activity against multiple CoVs in animal studies^[^ ^2^ ^]^ and is currently in Phase II clinical trials on symptomatic patients with COVID‐19.^[^ ^3^ ^]^ This antiviral candidate is structurally similar to remdesivir, but blocks RNA polymerase differently, which possibly makes them complimentary.^[^ ^4^ ^]^ Since EIDD‐2801 can be taken orally in the form of a pill, the potential treatment against COVID‐19 would be made more accessible compared to remdesivir that currently has to be administered intravenously.

EIDD‐2801 was developed by Emory University researchers, and the disclosed route consists of a five‐step synthesis starting from uridine (Scheme 1a).^[^ ^5^ ^]^ This was the only reported synthesis in the open literature until recently, when routes toward EIDD‐2801 were reported that replaced uridine for cytidine.^[^ ^6^, ^7^ ^]^ In the original route, first an acetonide protection followed by selective esterification of the 5'‐hydroxy group is performed in a one‐pot fashion. Then, the molecule is activated by introduction of the 1,2,4‐triazole moiety which is further displaced by hydroxylamine. After removal of the acetonide protecting group, EIDD‐2801 is obtained via a complex crystallization procedure. As no isolated yield for the final deprotection step has been disclosed in the patent, an overall yield (17 %) can only be stated for the synthesis of protected EIDD‐2801. The main disadvantage of the reported route is the low yield (29 %) in the triazole coupling step.

**Scheme 1:**
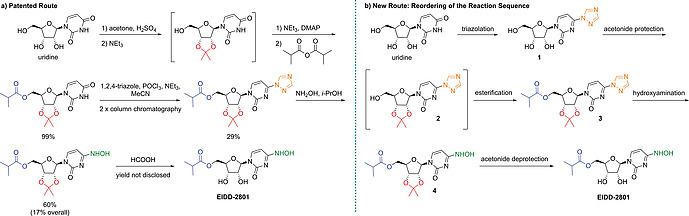
Patented route (a) vs. proposed route (b) toward EIDD‐2801.

In 1997, Reese and co‐workers reported the triazolation of uridine to proceed in 89 % yield after a simple extractive work‐up and crystallization.^[^ ^8^ ^]^ Therefore, we anticipated, that a simple reordering of the steps in the original synthetic sequence would result in an overall higher yield and improved isolation procedures. Further, by starting from uridine the impurity profile should closely match that of the commercial process, which might accelerate uptake of the modified process by minimizing regulatory changes. As illustrated in Scheme 1b, we planned our synthesis toward EIDD‐2801 starting with the triazolation according to Reese and co‐workers. The other transformations (acetonide protection, esterification, hydroxyamination) should ideally proceed in a similar approach as in the patented route. Acetonide deprotection from **4** as final step and isolation of EIDD‐2801 is envisaged as reported in the original patent.^[^ ^5^ ^]^

Following the original procedure of Reese and co‐workers, which involves a one‐pot TMS protection of uridine and phosphorus oxychloride‐promoted triazole coupling using Et_3_N as base,^[^ ^8^ ^]^ **1** was obtained in 74 % yield. When changing the base to *N*‐methylpyrrolidine the yield of **1** increased to 88 % (Scheme 2). Product isolation consisted of a simple filtration/washing step, as **1** precipitated during deprotection from the MeOH/AcOH mixture. Additionally, we attempted to reduce the amount of 1,2,4‐triazole (10 equiv.), because of the associated health risks.^[^ ^9^ ^]^ Unfortunately, less triazole also leads to lower isolated yields of **1** (8 equiv.: 78 %, 6 equiv.: 58 %). Compared to the original route,^[^ ^5^ ^]^ we were able to increase the yield for the triazolation step from 29 % to 88 % by introducing the triazole moiety as first step. Importantly, also column chromatographic work‐up could be avoided.

**Scheme 2:**
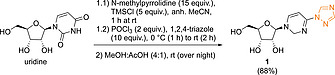
Step 1: Triazolation of uridine.

To selectively introduce the isobutyl ester at the 5'‐hydroxy position, the other two hydroxy groups need to be protected first. When using the conditions for the acetonide protection of uridine described in the original patent (5 mol‐% H_2_SO_4_ in acetone),^[^ ^5^ ^]^ no conversion of **1** to the desired acetonide **2** was observed. Higher concentrations (0.5 equiv.) of H_2_SO_4_ lead to hydrolysis of the triazole moiety. Addition of 2 equiv. of 2,2‐dimethoxypropane (DMP) did not significantly improve the reaction. However, when exchanging acetone for MeCN as solvent in combination with the use of DMP, a 73 % conversion to **2** was achieved. Further optimization revealed, that the amount of H_2_SO_4_ could be reduced to 5 mol‐% (Scheme 3).

**Scheme 3:**
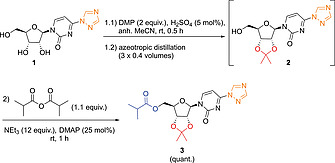
Steps 2 and 3: One‐pot acetonide protection and esterification. See the Supporting Information for more details.

Upon attempting the acetonide formation and esterification in a one‐pot fashion similar to the patented route (see Scheme 1a1), we observed that 3 equiv. of isobutyric anhydride were needed in order to reach full conversion of **2**. The excess of anhydride was necessary, because it was quenched by the 2 equiv. of MeOH that were released from DMP. Therefore, after stirring of **1** with DMP and H_2_SO_4_ in MeCN for 30 min at room temperature, a stepwise azeotropic distillation was performed to remove MeOH. Applying this protocol resulted in only 1.1 equiv. of isobutyric anhydride being necessary for full conversion of **2** (Scheme 3). A single distillation step of 1.2 volumes proved to be less efficient, as 1.3 equiv. of anhydride were required. Acetonide ester **3** was obtained in quantitative yield and ≥ 99 % HPLC purity after extractive work‐up in this one‐pot procedure.

Acetonide ester **3** can be readily converted to hydroxylamine **4** by stirring in *i*PrOH at room temperature with 1.5 equiv. of hydroxylamine (50 wt.‐% in H_2_O) for 20 min (Scheme 4). After extractive work‐up, **4** was isolated in 90 % yield and 91 % HPLC purity. The purity could be improved to 99 % by washing with cold diethyl ether, but ca 50 % of product was lost in this washing step. Therefore, the washing step was omitted, and the crude product after extraction was used for the acetonide deprotection step.

**Scheme 4:**
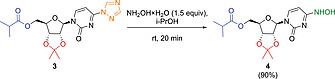
Step 4: Hydroxylamine formation.

Removal of the acetonide protecting group of **4** leading to EIDD‐2801 has been described to proceed in formic acid at room temperature overnight (see Scheme 1a).^[^ ^5^ ^]^ However, by performing the reaction exactly under the reported conditions only a 47 % conversion to EIDD‐2801 was obtained. Heating to 60 °C for 5 h or 100 °C for 30 min resulted in the increased formation of side products. An acid screening (see Table S2) revealed that H_2_SO_4_ in combination with *i*PrOH as solvent furnished the highest conversion (80 %) to EIDD‐2801 after heating at 60 °C for 30 min. The main side reaction was found to be the ester hydrolysis yielding *N*‐hydroxycytidine **5** (EIDD‐1931, see also Figure 1), in particular when the reaction was conducted at higher temperatures.

**Figure 1:**
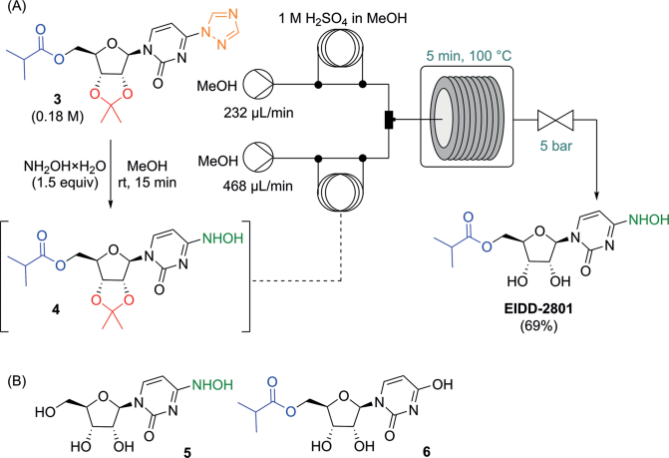
Steps 4 and 5: Telescoped batch hydroxyamination and continuous flow acetonide deprotection. (A) Schematic representation of the reaction set‐up. (B) Structures of identified side products.

Since the same solvent system (*i*PrOH) was employed in the hydroxyamination and acetonide deprotection, we envisioned that these two steps could be combined to a one‐pot procedure. A similar process has been recently demonstrated in an alternative route toward EIDD‐2801.^[^ ^7^ ^]^ During batch optimization studies (see Table S3) we experienced reproducibility issues that were mainly related to the exothermic nature upon H_2_SO_4_ addition in the deprotection step. Therefore, we decided to transfer the batch process to continuous flow, where better mixing and temperature control allows a superior control of exotherms and in return would provide reproducible results.^[^ ^10^, ^11^, ^12^ ^]^

A simple flow set‐up was therefore established using a heated reaction coil together with individual streams for introducing solutions of the substrate and the acid reagent, which were combined in a Y‐shaped mixer (Figure 1A). Triazole **3** was converted to hydroxylamine **4** in batch under the same conditions as shown in Scheme 4. However, **4** was not isolated, but after 15 min of stirring, the reaction mixture was introduced directly into the flow reactor via a sample loop for the acetonide deprotection. The flow process was investigated in various solvents using HCOOH, H_2_SO_4_, CF_3_COOH or TfOH, respectively, as acid component (Tables S4–S7). During the deprotection optimization studies, ester hydrolysis and hydroxylamine–hydroxyl exchange occurred as most prominent side reactions yielding compounds **5** and **6** (Figure 1B, see also the Supporting Information). The effects of reaction temperature, residence time and excess of acid were therefore carefully examined with the aim to minimize side product formation while simultaneously maximize conversion to EIDD‐2801 (see Tables S4–S7 for details). Similarly as in batch, H_2_SO_4_ proved to be superior to the other tested acids (see Table S5). Because of precipitation issues in *i*PrOH, MeOH was selected as solvent. The highest conversion to EIDD‐2801 (79 %) was obtained at only 5 min of residence time using 2.75 equiv. of H_2_SO_4_ at 100 °C, while **5** (11 %) and **6** (8 %) were at a minimum. Importantly, the flow process under optimum conditions not only permitted higher yields, but it also enabled a significant chemical intensification and a proper reproducibility as compared to our initial batch attempts. By employing these flow conditions for scale‐out (16 min), pure EIDD‐2801 was obtained in 69 % isolated yield (307 mg) after chromatographic purification.

In conclusion, an improved protocol was developed to access EIDD‐2801 from uridine as starting material (Scheme 5). By strategically reordering of synthetic steps and by employing a continuous flow process for the final acetonide deprotection, the overall yield was improved from 17 % to 61 %. Importantly, this strategy presents fewer and simplified product isolation procedures, as two telescoped procedures (acetonide protection/esterification and hydroxyamination/acetonide deprotection) are included within the 5‐step route.

**Scheme 5:**
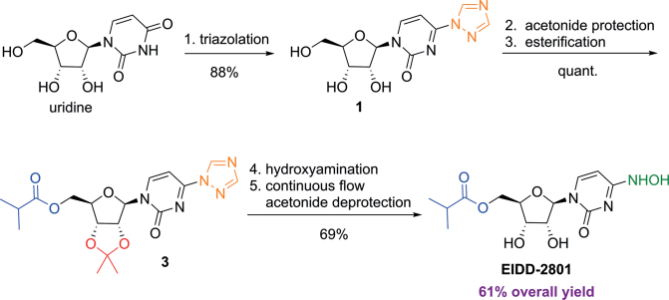
Improved protocol for the synthesis of EIDD‐2801.

## Supporting information

Supporting Information
