# Supplementary material for: A High‐Yielding Synthesis of EIDD‐2801 from Uridine
Source: European J Org Chem. 2020 Nov 12;2020(43):6736–9. doi: 10.1002/ejoc.202001340 (PMC7894511; doi:10.1002/ejoc.202001340)
Supplement: Supplementary file 1 — Supporting Information [file EJOC-2020-6736-s001.pdf]

European Journal of Organic Chemistry

Supporting Information

A High-Yielding Synthesis of EIDD-2801 from Uridine**

Alexander Steiner, Desiree Znidar, Sándor B. Ötvös, David R. Snead, Doris Dallinger,* C. Oliver Kappe*

Contents

1. General Methods S2

2. Optimization Studies: One-pot Acetonide Protection and Esterification S3

3. Hydroxylamine Formation S6

4. Optimization Studies: Acetonide Deprotection in Batch S7

5. Optimization Studies: Acetonide Deprotection in Continuous Flow S9

6. Acetonide Deprotection: Analysis of Side Products S12

7. Experimental Procedures S13

7.1. Synthesis of Triazolated Uridine **1** S13

7.2. One-pot Synthesis of Acetonide Ester **3** S13

7.3. Synthesis of Acetonide-protected Hydroxylamine **4** S14

7.4. Telescoped Synthesis of **EIDD-2801** in Continuous Flow S15

8. NMR Spectra S16

9. References S24

1. General Methods

All solvents and chemicals were obtained from standard commercial vendors (TCI, Sigma-Aldrich/Merck or VWR) and were used without any further purification, unless otherwise noted. **^{1}H NMR spectra** were recorded on a Bruker 300 MHz instrument. **^{13}C NMR spectra** were recorded on the same instrument at 75 MHz. Chemical shifts (δ) are expressed in ppm downfield from TMS as internal standard. The letters s, d, t, q, sept, dd and m are used to indicate singlet, doublet, triplet, quadruplet, septet, doublet of doublets and multiplet. **Analytical HPLC analysis** was carried out on a Shimadzu instrument using a C18 reversed-phase (RP) analytical column (150 mm × 4.6 mm, particle size 5 µm) at 37 °C using mobile phases A (H_2O/MeCN (90:10 v/v) + 0.1% TFA) and B (MeCN + 0.1% TFA) at a flow rate of 1.5 mL/min. The following gradient was applied: start at 3 % solvent B, increase to 5 % solvent B until 3 min, increase to 30 % solvent B until 7 min and finally increase to 100 % solvent B until 10 min. **LC-MS analysis** was carried out on a Shimadzu instrument using a C18 reversed-phase (RP) analytical column (150 mm × 4.6 mm, particle size 5 µm) using mobile phases A (H_2O/MeCN 90:10 (v/v) + 0.1% HCOOH) and B (MeCN + 0.1 % HCOOH) at a flow rate of 0.6 mL/min. The following gradient was applied: hold at 5% solvent B until 2 min, increase to 20% solvent B until 8 min, increase to 100% solvent B until 16 min and hold until 22 min at 100% solvent B. Low resolution mass spectra were obtained on a Shimadzu LCMS-QP2020 instrument using electrospray ionization (ESI) in positive or negative mode. **Melting points** were obtained on a Stuart melting point apparatus in open capillary tubes. **High-resolution mass spectrometry** was performed on an Agilent 6230 TOF mass spectrometer, after separation of the compounds with an Agilent 1260 Infinity Series HPLC-system. The injection volume was set to 0.5 µL and the flow rate to 0.3 mL/min of a mixture of 40% H_2O (0.1% 5M ammoniumformate) and 60% MeCN/H_2O (5:1 +0.1% 5 M ammoniumformate). The HRMS module comprises an electrospray ionization source (Dual AJS ESI) and uses nitrogen as the nebulizer (15 psig) and the drying gas (5 L/min). ESI experiments were performed using the positive ionization mode (Gas Temp. = 300 °C, Fragmentor = 150 V, Skimmer = 65 V, OCT 1 RF Vpp = 750 V, Vcap = 1400, Nozzle Voltage = 2000 V, Reference Masses = 121.050873 and 922.009798, Acquisition = 100-1,100 m/z, 1 spectra/s). Data was acquired with MassHunter Workstation Rev.B.05.01SP2. **Microwave irradiation** experiments were carried out in a Monowave 400 single-mode microwave reactor from Anton Paar or an Initiator+ single-mode microwave reactor from Biotage, respectively, using 10 mL Pyrex vials. The reaction temperature was controlled by an external infrared sensor. Reaction times refer to hold times at the temperature indicated. **Column chromatography** was carried out using a Biotage Isolera automated flash chromatography system.

2. Optimization Studies: One-pot Acetonide Protection and Esterification

Table S1. Optimization of Acetonide Formation.

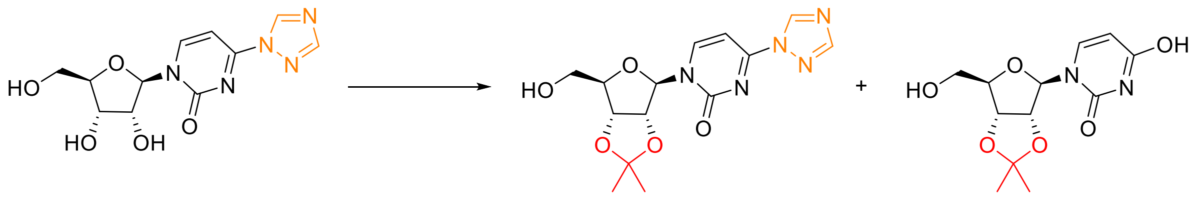

1 **2** **7**

Entry	Solvent	Reagent[a]	Time [h]	**1** [%][b]	**2** [%][b]	**7** [%][b]
1	acetone	H_2SO_4 (0.5)	24	1	3	96
2	acetone	H_2SO_4 (0.2) DMP (2)	4	78	12	10
3	MeCN	H_2SO_4 (0.2) DMP (2)	1	23	73	4

[a] Equivalents given in parenthesis.
[b] Area% determined by HPLC at 260 nm.

The hydrolysis product **7** was identified by LC-MS analysis (see Figure S1). The generation of **7** was additionally verified, since upon reaction with isobutyric anhydride, the corresponding ester **8** was obtained. The identity of **8** was confirmed by NMR analysis (see Figures S2 and S3).

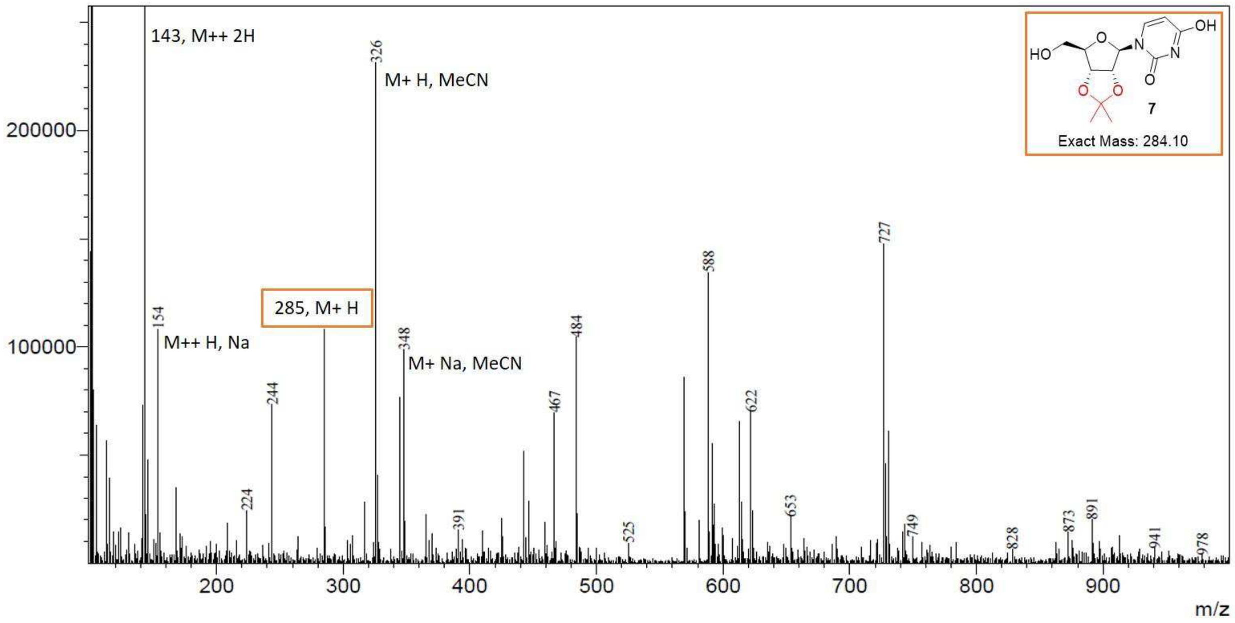

Figure S1. LC-MS of hydrolysis product **7.**

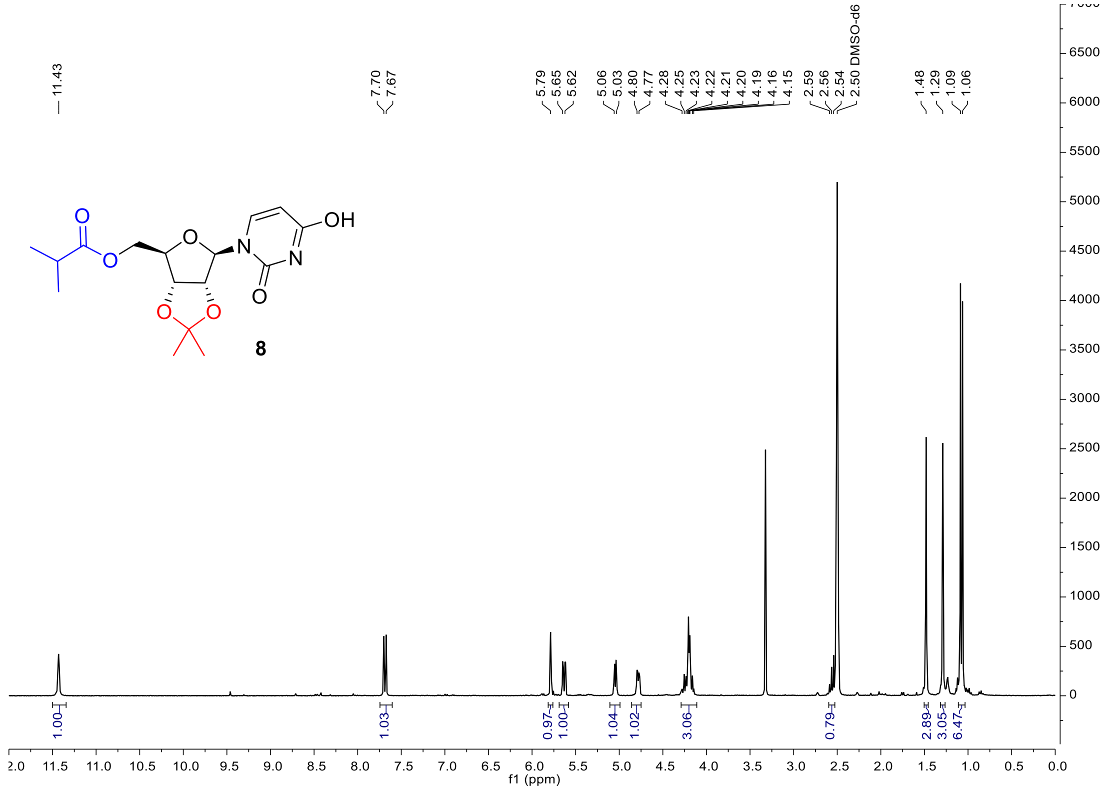

Figure S2. ^{1}H NMR (300 MHz, DMSO-d_6) of product **8.**

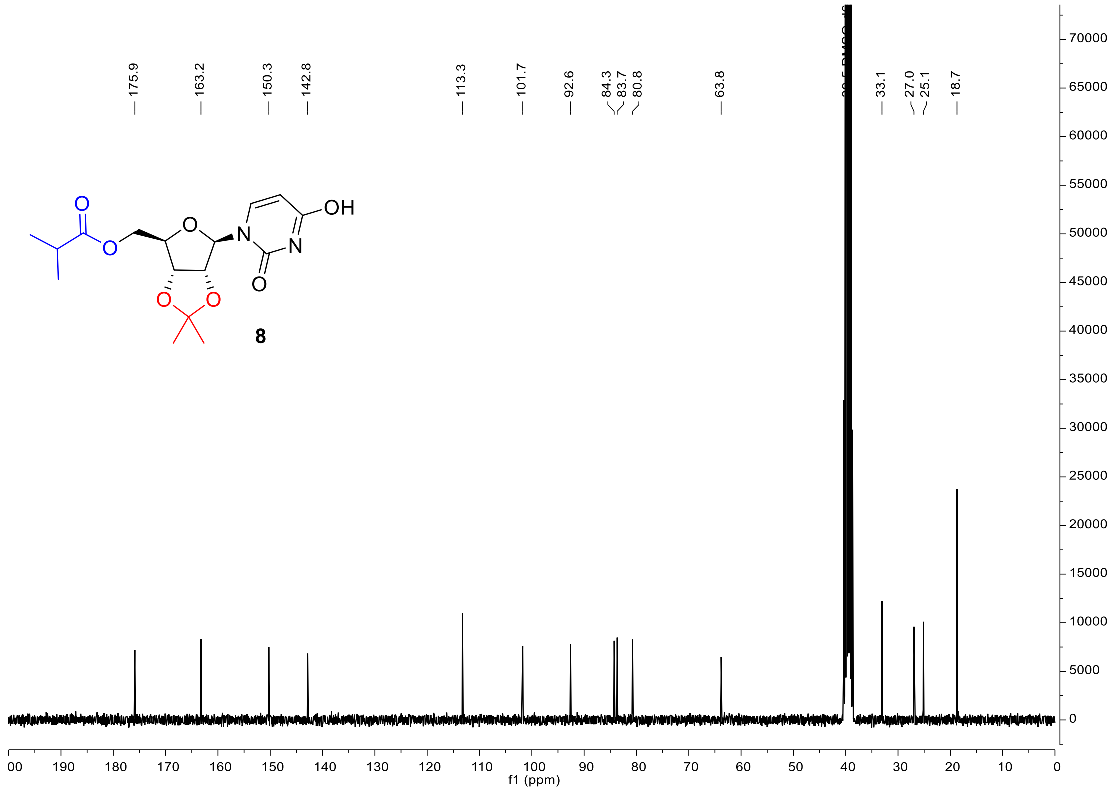

Figure S3. ^{13}C NMR (75 MHz, DMSO-d_6) of product **8.**

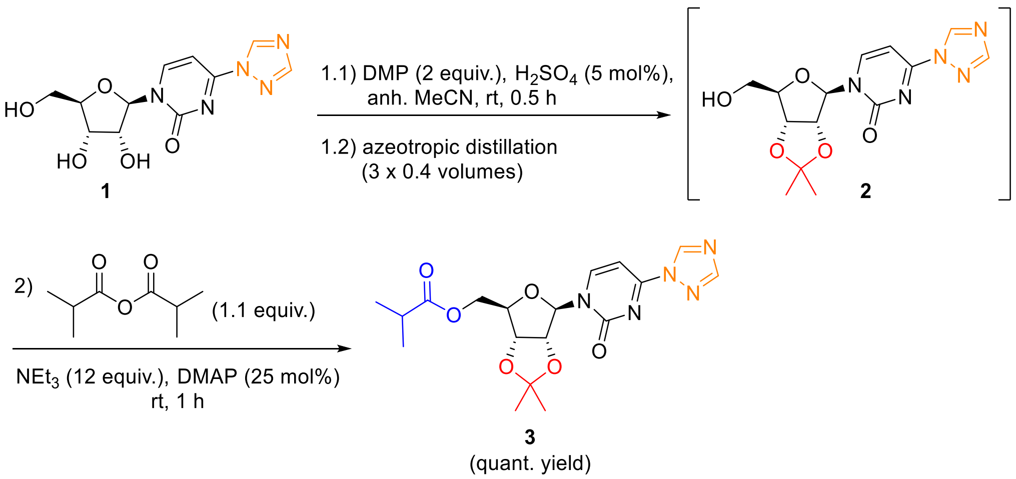

Scheme S1. One-pot acetonide protection and esterification.

In the protection step, the reaction mixture was stirred first for 30 min at rt, then the stepwise azeotropic distillation was performed. We chose this reaction regime out of precautionary reasons, because the bp of DMP (85 °C) is close to the bp of the azeotrope MeOH/MeCN (63 °C). Therefore, in order not to potentially distill DMP during the azeotropic distillation, initially stirring at rt was preferred. A 73% conversion to **2** was achieved after 30 min, and full conversion was accomplished during the azeotropic distillation (Figure S4). Reaction monitoring of the esterification step could be done visually, as with full conversion of **2**→**3** the suspension became a clear solution. The reaction time was dependent on the scale: On a 600 mg scale the reaction went to completion within 40 min (Figure S4), while on a 5 g scale the reaction time needed to be prolonged to 1 h (Scheme S1). In general, the reaction sequence proved to be remarkably clean, as no side products were detected. After extractive work-up, **3** was isolated in quantitative yield and a purity of ≥99%.

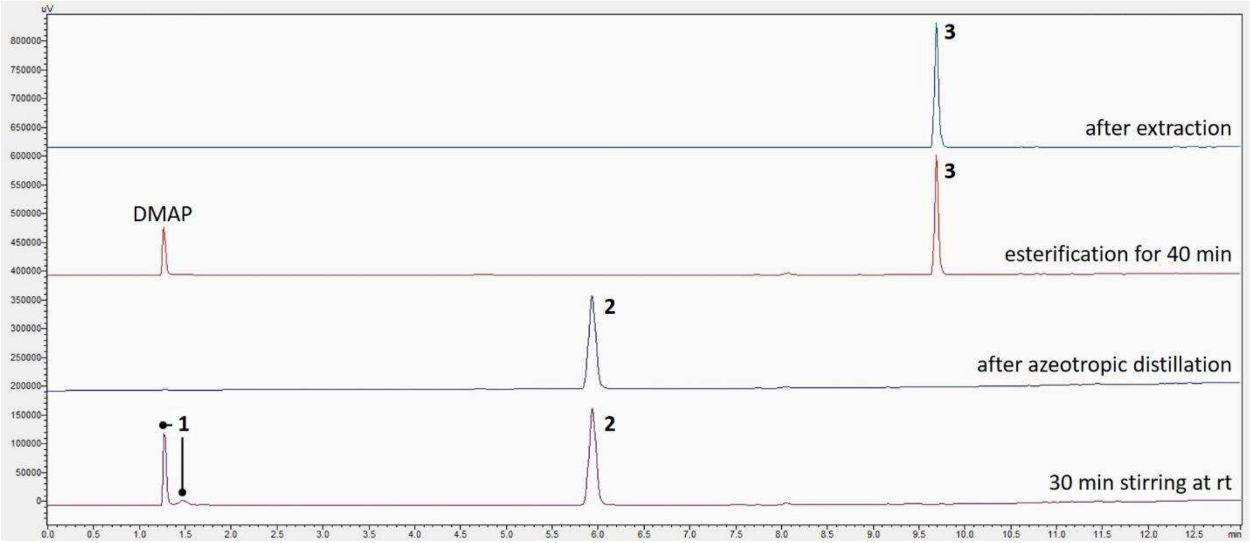

Figure S4. HPLC (260 nm) reaction monitoring of the one-pot acetonide protection and esterification on a 600 mg scale.

3. Hydroxylamine Formation

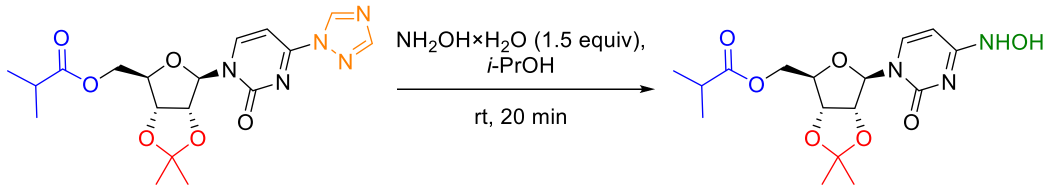

3 **4**

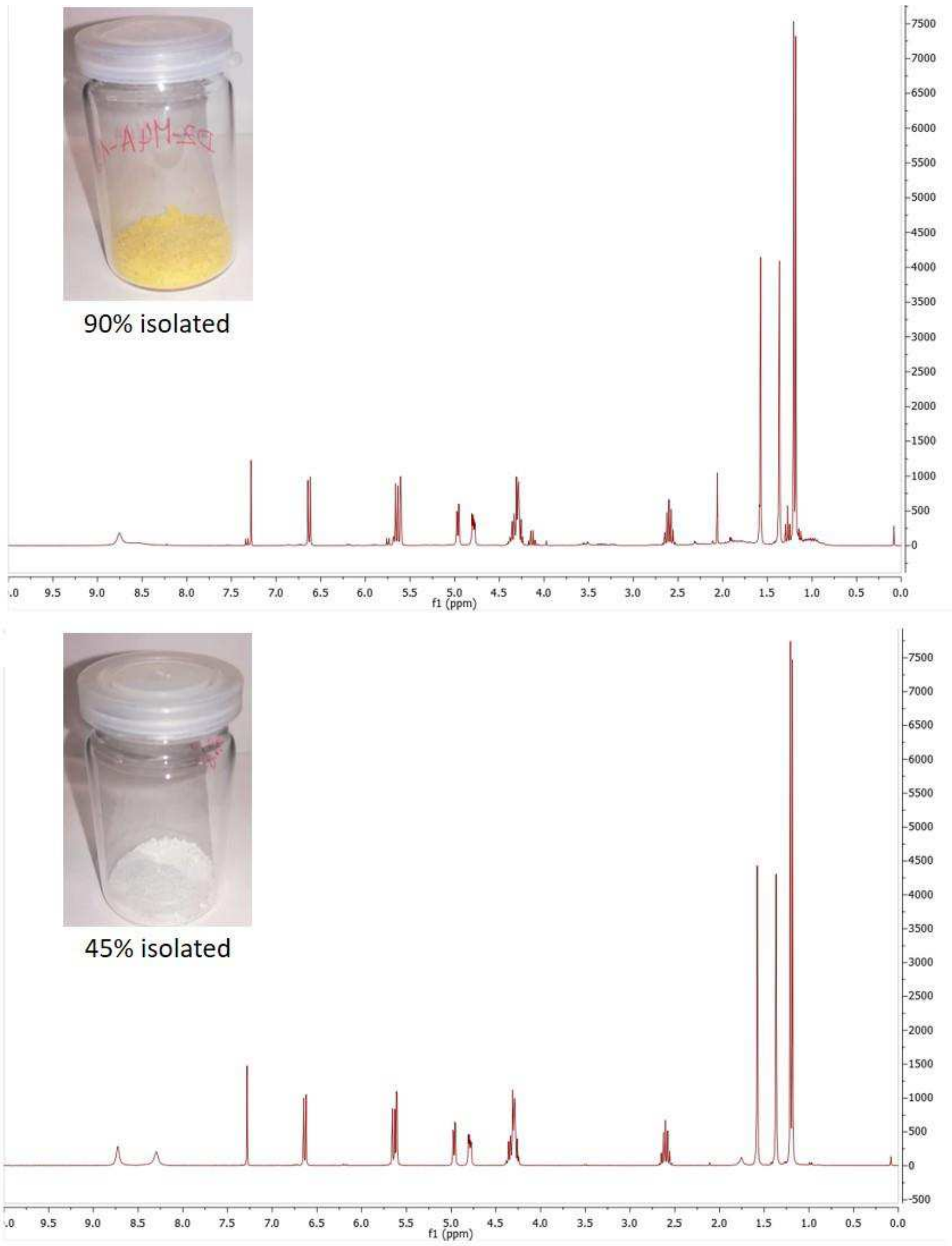

Figure S5. ^{1}H NMR comparison of isolated product **4** before (top) and after (bottom) the washing step with Et_2O.

4. Optimization Studies: Acetonide Deprotection in Batch

General procedure for the acid screening: A 1 mL HPLC vial was charged with **4** (50 mg, 0.135 mmol) and 1 mL of acid. The vial was crimped and stirred at rt or heated in an aluminum heating block at 60 °C.

Table S2. Acid Screening for the Acetonide Deprotection of **4**.

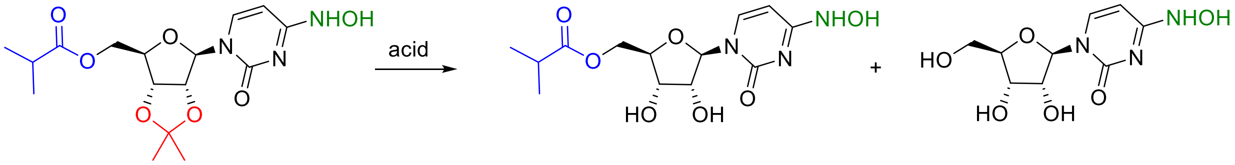

4 **EIDD-2801** **5**

Entry	Acid	T [°C]	Time [h]	**4** [%][b]	**EIDD-2801** [%][b]	**5** [%][b]
1	HCOOH	rt	22	40	**47**	-
2	HCOOH	60	5	6	**33**	-
3	H_2SO_4 (*i*-PrOH)[a]	rt	7	28	**62**	5
4	H_2SO_4 (*i*-PrOH)[a]	60	0.5	10	**80**	8
5	H_2SO_4 (*i*-PrOH)[a]	60	1	8	**74**	15
6	H_2SO_4 (H_2O)[a]	rt	1	40	**52**	4
7	H_2SO_4 (H_2O)[a]	60	0.5	-	**43**	56
8	HCl_{conc} (*i*-PrOH)[a]	rt	7	55	**32**	6
9	HCl_{conc} (*i*-PrOH)[a]	60	0.5	-	**36**	64
10	HCl conc	rt	1	33	**59**	5

[a] Acids were 1 M in the respective solvent.
[b] Area% determined by HPLC at 260 nm. Except for entry 9, further unidentified impurities were detected.

General procedure for the one-pot hydroxyamination and acetonide deprotection: A microwave vial was charged with **3** (50 mg, 0.123 mmol). *i*-PrOH (617 µL) and hydroxylamine (50w% in water, 11.1 µL, 1.5 equiv.) were added and the reaction mixture was stirred at room temperature for 20 minutes to ensure full conversion to **4**. Next, conc H_2SO_4 was added dropwise under stirring, the microwave vial crimped and subjected to microwave heating.

As can be seen in Table 3 (entries 1 and 2), the preformation of hydroxylamine **4** is required in order to drive the reaction toward EIDD-2801 while concomitantly reduce the formation of side products **5** and **6**. Nevertheless, we experienced reproducibility issues in this optimization study, most likely because of the exotherm upon addition of H_2SO_4, which proved to be difficult to control in batch. Unfortunately, the reproducibility could not be improved by diluting the conc H_2SO_4 with *i*-PrOH.

Table S3. Optimization for the One-pot Hydroxyamination and Acetonide Deprotection.

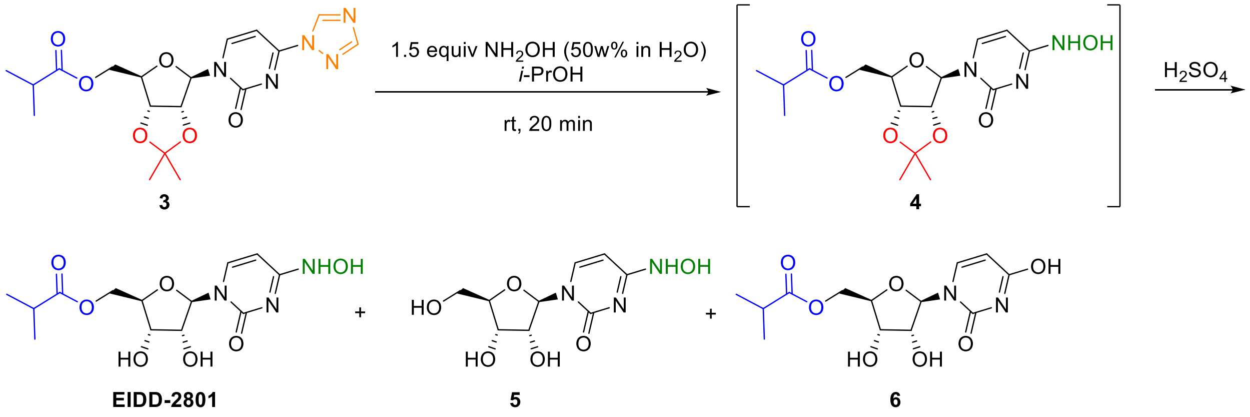

Entry	Equiv H_2SO_4	T [°C][a]	Time [h]	**4** [%][b]	**EIDD-2801** [%][b]	**5** [%][b]	**6** [%][b]
1[c]	5	60	0.5	-	**9**	29	62
2[c]	5	60	1	-	**7**	44	49
3	5	60	0.5	1	**71**	12	12
4	5	40	0.5	30	**53**	3	8
5	1.5	60	0.5	80	**7**	-	-
6	6	60	0.5	25	**57**	5	7
7	10	rt	1	16	**68**	3	7
8	10	rt	2	12	**75**	5	8
9	10	rt	4	6	**72**	10	8

[a] Close-vessel microwave heating for T ≥40 °C.
[b] Area% determined by HPLC at 260 nm. Except for entries 1, 2 and 8, further unidentified impurities were detected.
[c] No preformation of **4**.

5. Optimization Studies: Acetonide Deprotection in Continuous Flow

The flow set-up consisted of a 3.5 mL reaction coil (PFA, 1/16" OD, 0.80 mm ID) immersed in a heated oil bath, a Syrris Asia syringe pump module (P1 and P2) equipped with two injection valves, two sample loops (SL1 and SL2, 2 mL and 1.5 mL, respectively; PFA, 1/16" OD, 0.80 mm ID, each) and a Zaiput back pressure regulator which kept a constant pressure of 5 bar.

General procedure: For each experiment, a 2 mL solution containing 0.18 M of compound **3** (146 mg, 0.36 mmol) and 1.5 equiv. of NH_2OH (32.4 µL, 50 wt% in H_2O) in the corresponding solvent was stirred for 15 min at room temperature to ensure full conversion to **4**. This reaction mixture was then directly transferred to SL2. SL1 was filled with neat HCOOH or with a solution of H_2SO_4, CF_3COOH or TfOH prepared in the same solvent as the substrate solution. The liquid feeds were combined in a Y-mixer, and the resulting stream was directed through the heated reaction coil. In each run, approx. 0.5 mL sample of product mixture was collected which was next analyzed directly by HPLC at 260 nm.

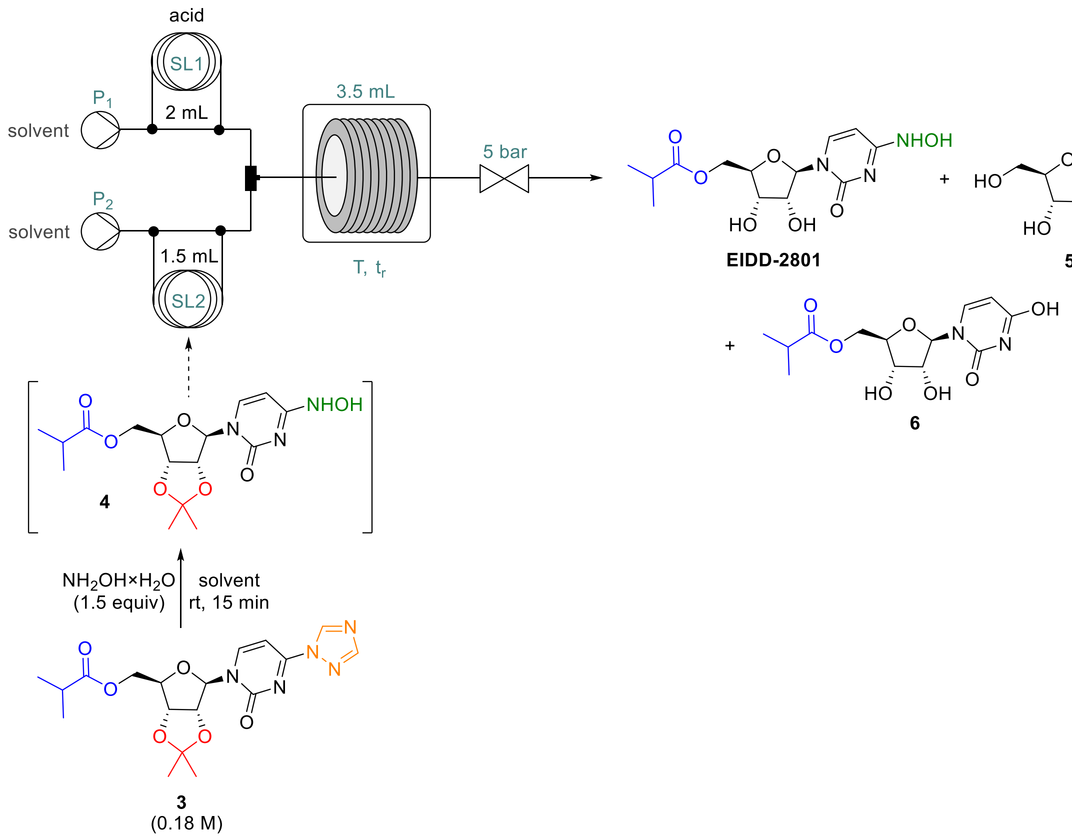

Figure S6. Flow set-up used for the optimization experiments.

Table S4. Optimization of the Flow Acetonide Deprotection using **HCOOH** as Acid Reagent.[a]

Entry	Solvent	Flow rate [μL/min]		Acid equiv	t_r [min]	T [°C]	**4** [%][b]	**EIDD-2801** [%][b]	**5** [%][b]	**6** [%][b]
		P1	P2							
1	*i*-PrOH	175	175	144	10	60	95.8	**4.2**	-	-
2	MeOH	175	175	144	10	60	94.0	**6.0**	-	-
3[c]	MeOH	106	69	222	20	100	11.3	**71.7**	1.4	6.4

[a] Neat HCOOH (≥98%) was employed which corresponds to approx. 26 M.
[b] Area% determined by HPLC at 260 nm.
[c] Further unidentified impurities.

Table S5. Optimization of the Flow Acetonide Deprotection using **H_2SO_4** as Acid Reagent.[a]

Entry	Solvent	Flow rate [μL/min]		Acid equiv	t_r [min]	T [°C]	**4** [%][b]	**EIDD-2801** [%][b]	**5** [%][b]	**6** [%][b]
		P1	P2							
1[c]	*i*-PrOH	175	175	5.55	10	60	56.3	**38.3**	1.0	4.5
2[c]	THF	175	175	5.55	10	60	-	**-**	-	-
3	MeOH	175	175	5.55	10	60	6.7	**73.5**	13.6	6.2
4	MeOH	58	117	2.75	20	60	24.6	**60.8**	9.5	5.1
5	MeOH	500	250	11.1	5	60	3.0	**72.9**	18.6	5.5
6	MeOH	232	468	2.75	5	80	38.2	**54.6**	3.4	3.9
7	MeOH	116	234	2.75	10	80	7.3	**73.7**	12.0	7.0
8	MeOH	350	350	5.55	5	100	-	**46.2**	47.6	6.2
9	MeOH	232	468	2.75	5	100	1.8	**79.1**	11.0	8.1
10	MeOH	116	234	2.75	10	100	-	**55.7**	36.2	8.1
11	MeOH	80	270	1.65	10	100	5.4	**70.2**	16.8	7.6
12	MeOH	342	534	3.55	4	100	-	**70.3**	19.7	10.0
13	MeOH	167	417	2.22	6	100	3.7	**75.9**	11.9	8.5
14	EtOH	232	468	2.75	5	100	3.7	**73.6**	7.0	15.7
15[d]	MeCN	232	468	2.75	5	100	9.4	**17.1**	2.1	62.6
16	MeOH/H_2O 8:1	232	468	2.75	5	100	2.3	**70.8**	10.3	16.5
17	MeOH/H_2O 4:1	232	468	2.75	5	100	1.8	**67.0**	10.5	20.8

[a] H_2SO_4 was employed as 1 M solution in MeOH.
[b] Area% determined by HPLC at 260 nm.
[c] Precipitation after mixing of streams.
[d] Further unidentified impurities.

Table S6. Optimization of the Flow Acetonide Deprotection using **CF_3COOH** as Acid Reagent.

Entry	Solvent	Flow rate [µL/min]		Acid equiv	t_r [min]	T [°C]	**4** [%][a]	**EIDD-2801** [%][a]	**5** [%][a]	**6** [%][a]
		P1	P2							
1[b]	MeOH	232	468	2.75	5	100	89.7	**10.3**	0	0
2[b]	MeOH	175	175	5.55	10	100	76.6	**23.4**	0	0
3[c]	MeOH	156	80	70	15	100	10.4	**61.9**	11.7	16.0

[a] Area% determined by HPLC at 260 nm.
[b] CF_3COOH was employed as 1 M solution in MeOH.
[c] CF_3COOH was employed as 6.5 M solution in MeOH.

Table S7. Optimization of the Flow Acetonide Deprotection using **TfOH** as Acid Reagent.[a]

Entry	Solvent	Flow rate [µL/min]		Acid equiv	t_r [min]	T [°C]	**4** [%][b]	**EIDD-2801** [%][b]	**5** [%][b]	**6** [%][b]
		P1	P2							
1	MeOH	116	234	2.75	10	80	6.7	**70.8**	14.5	8.0
2	MeOH	232	468	2.75	5	100	0.9	**73.7**	17.7	7.6

[a] TfOH was employed as 1 M solution in MeOH.
[b] Area% determined by HPLC at 260 nm.

6. Acetonide Deprotection: Analysis of Side Products

In the acetonide deprotection reactions, ester hydrolysis and the exchange of the hydroxyl amine moiety occurred as most prominent side reactions yielding compounds **5** and **6** as side products as detailed above. The identity of these substances was corroborated by means of LC-MS.

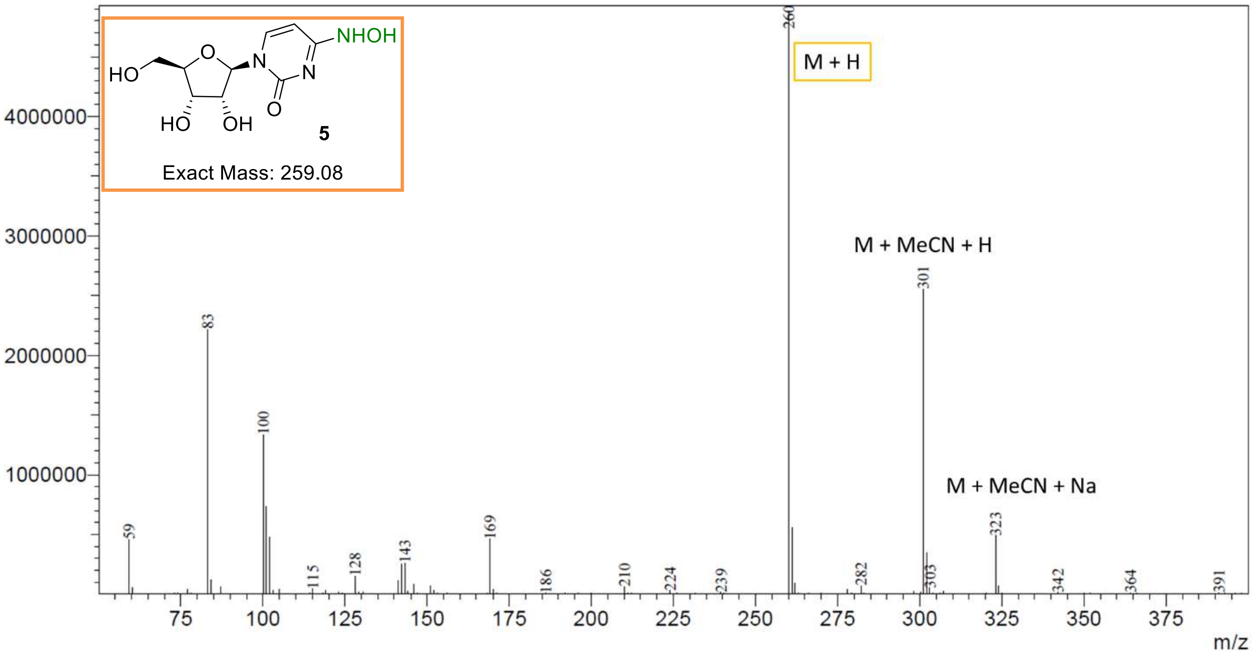

Figure S7. LC-MS of compound **5**.

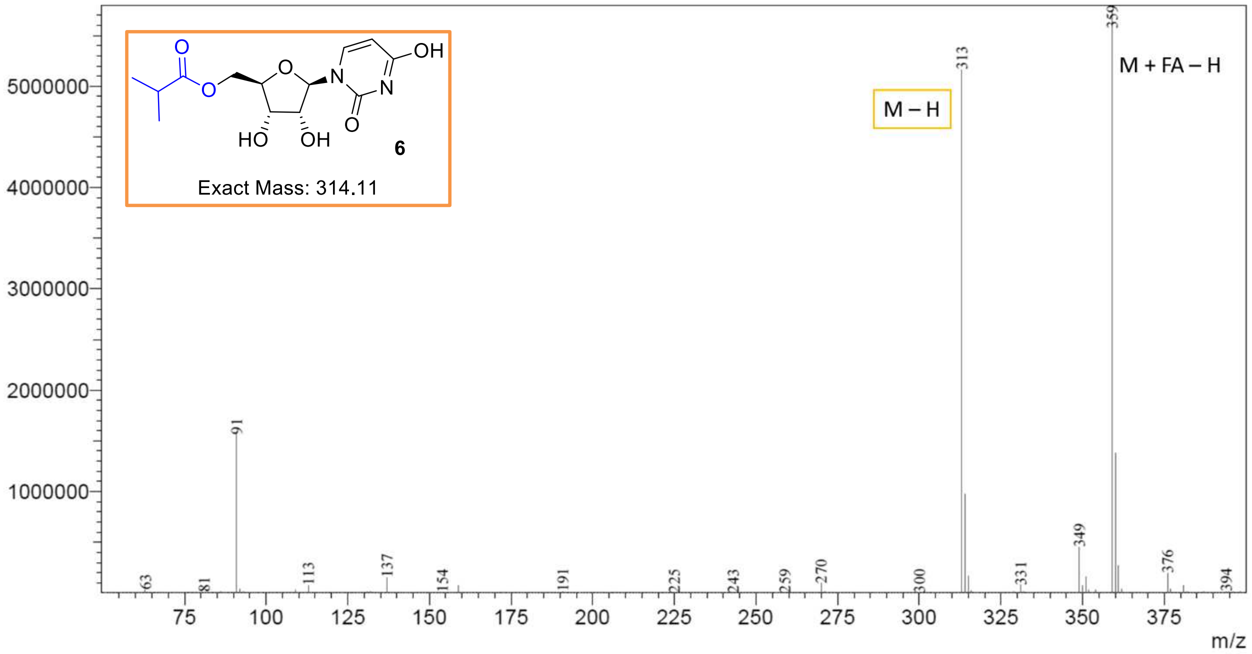

Figure S9. LC-MS of compound **6** (FA: formic acid).

7. Experimental Procedures

7.1. Synthesis of Triazolated Uridine 1

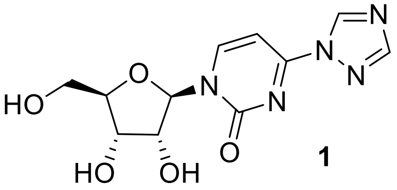

1

An oven dried 500 mL 2-necked round bottom flask was flushed with argon and charged with uridine (5.0 g, 20.5 mmol) and 164 mL of anhydrous MeCN (AcroSeal^{TM}, max. 0.001% H_2O). *N*-Methylpyrrolidine (31.9 mL, 307 mmol, 15 equiv) and TMSCl (13 mL, 102 mmol, 5 equiv) were added, and the reaction mixture was allowed to stir for 1 h at room temperature. The solution was cooled in an ice bath to 0 °C. $POCl_3$ (3.74 mL, 41.0 mmol, 2 equiv) was added. After stirring for 10 min, 1,2,4-triazole (14.1 g, 205 mmol, 10 equiv) was added, and stirring was continued at 0 °C for 1 h and at room temperature for another 2 h. The yellow solution was then poured onto 700 mL triethylammonium phosphate buffer (0.5 M, pH 7) and extracted with DCM (3 × 100 mL). The combined organic phases were dried over Na_2SO_4 and the solvent evaporated under reduced pressure. A mixture of MeOH:AcOH (4:1 v/v) was added to the residue and the reaction mixture was stirred at room temperature overnight. The precipitated product was collected by filtration, washed with diethyl ether and dried under reduced pressure. **1** was obtained as a white solid in 88% yield (5.34 g) and ≥99% purity.

^{1}H NMR (300 MHz, DMSO-d_6) δ 9.45 (s, 1H), 8.84 (d, *J* = 7.2 Hz, 1H), 8.41 (s, 1H), 6.97 (d, *J* = 7.2 Hz, 1H), 5.80 (s, 1H), 5.65 (d, *J* = 4.6 Hz, 1H), 5.26 (t, *J* = 4.5 Hz, 1H), 5.06 (d, *J* = 5.2 Hz, 1H), 4.05 – 3.97 (m, 3H), 3.81 (dd, *J* = 11.6, 4.0 Hz, 1H), 3.64 (dd, *J* = 12.3, 3.0 Hz, 1H). ^{13}C NMR (75 MHz, DMSO-d_6) δ158.6, 154.1, 153.8, 148.4, 143.7, 93.7, 91.2, 84.2, 74.6, 68.1, 59.4.

The NMR data is in agreement with previously published values.[1]

7.2. One-pot Synthesis of Acetonide Ester 3

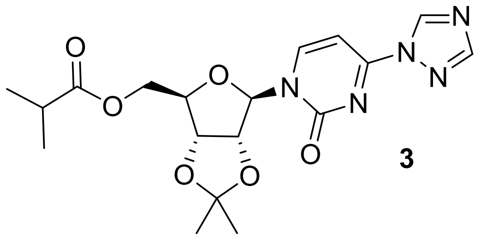

3

An oven dried 500 mL 3-necked round bottom flask equipped with a Dean-Stark apparatus was flushed with argon and charged with **1** (5.34 g, 18.1 mmol) and 121 mL of anhydrous MeCN. 2,2-Dimethoxypropane (4.46 mL, 36.2 mmol, 2 equiv) and 95% H_2SO_4 (53.4 µL, 0.91 mmol, 5 mol%) were added. The suspension was stirred at room temperature for 30 min. Then, 45 mL of anhydrous MeCN were added and 45 mL of solvent were distilled off ($T_{oilbath}$ = 95 °C). This azeotropic distillation was performed three times. After cooling to room temperature, Et_3N (30.3 mL, 217 mmol, 12 equiv), *N*,*N*-

dimethylaminopyridine (553 mg, 4.53 mmol, 25 mol%) and isobutyric anhydride (3.3 mL, 19.9 mmol, 1,1 equiv) were added. The reaction mixture was stirred at room temperature for 1 h. The solvent was removed under reduced pressure. After addition of 270 mL ethyl acetate, the organic phase was washed with sat. $NaHCO_3$ (2 × 90 mL), water (90 mL) and brine (90 mL) and dried with Na_2SO_4. After removal of the solvent under reduce pressure, the product was obtained as a yellow solid in quantitative yield (7.43 g) and 101% yield).

^{1}H NMR (300 MHz, $CDCl_3$) δ 9.25 (s, 1H), 8.12 (s, 1H), 8.05 (d, *J* = 7.3 Hz, 1H), 7.04 (d, *J* = 7.2 Hz, 1H), 5.82 (d, *J* = 1.4 Hz, 1H), 5.02 (dd, *J* = 6.3, 1.5 Hz, 1H), 4.82 (dd, *J* = 6.3, 3.8 Hz, 1H), 4.54 – 4.50 (m, 1H), 4.38 – 4.36 (m, 2H), 2.50 (sept, *J* = 7.0 Hz, 1H), 1.59 (s, 3H), 1.37 (s, 3H), 1.13 (d, *J* = 7.0 Hz, 6H). ^{13}C NMR (75 MHz, $CDCl_3$) δ 176.5, 159.9, 154.3, 154.2, 147.8, 143.5, 114.6, 96.7, 94.8, 86.6, 85.5, 81.1, 64.1, 34.0, 27.2, 25.4, 19.1, 19.0. HRMS (ESI, positive mode): *m/z* $[M + H]^+$ Calcd for $[C_{18}H_{23}N_5O_6 + H]^+$: 406.1721, found: 406.1720.

7.3. Synthesis of Acetonide-protected Hydroxylamine 4

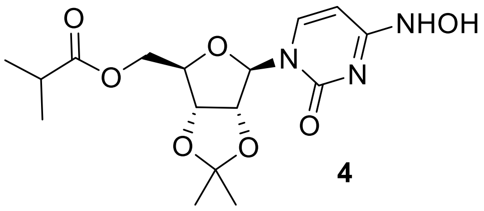

4

A 25 mL round bottom flask was charged with **3** (828 mg, 2.0 mmol) and 10 mL of *i*-PrOH. Hydroxylamine (50w% in H_2O, 185 µL, 3.0 mmol, 1.5 equiv) was added and stirred at rt for 20 min. Next, the solvent was removed under reduced pressure and the solid residue dissolved in 60 mL of EtOAc. The organic phase was washed with water (2 × 40 mL) and brine (1 × 40 mL) and dried over Na_2SO_4. After removal of the solvent under reduced pressure, the product was obtained as a yellow solid in 90% yield (682 mg) and 91% purity. The product was then further purified by washing with cold diethyl ether to obtain a white solid (341 mg, 45% yield, 99% purity).

^{1}H NMR (300 MHz, $CDCl_3$) δ 8.71 (brs, 1H), 8.27 (brs, 1H), 6.62 (d, *J* = 8.2 Hz, 1H), 5.64 – 5.58 (m, 2H), 4.95 (dd, *J* = 6.5, 2.1 Hz, 1H), 4.77 (dd, *J* = 6.4, 3.5 Hz, 1H), 4.40 – 4.18 (m, 3H), 2.58 (sept, *J* = 7.0 Hz, 1H), 1.56 (s, 3H), 1.35 (s, 3H), 1.18 (d, *J* = 7.0 Hz, 6H).^{13}C NMR (75 MHz, $CDCl_3$) δ 176.9, 149.4, 145.1, 132.9, 114.6, 98.9, 94.0, 84.6, 84.4, 81.2, 64.2, 34.0, 27.3, 25.5, 19.1, 19.1.

The NMR data is in agreement with previously published values.[2]

7.4. Telescoped Synthesis of EIDD-2801 in Continuous Flow

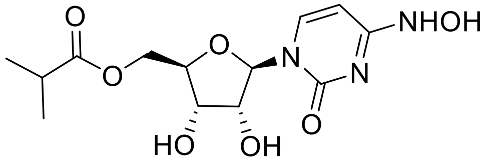

EIDD-2801

The flow set-up used was identical with the one described in Section 5 (Figure S6, see also Figure 1 in the main text). Sample loops of 5 mL (SL1) and 10 mL (SL2) were used (PFA, 1/16" OD, 0.80 mm ID, each).

Scale-out was performed under optimum flow conditions (see Table S5, entry 9) as follows:

A 10.5 mL solution containing 0.18 M of compound **3** (766 mg, 1.89 mmol) and 1.5 equiv. of NH_2OH (170 µL, 50 wt% in H_2O) in MeOH was prepared and was stirred for 15 min at room temperature. This reaction mixture was then directly transferred to SL2. SL1was filled with 1 M H_2SO_4 solution in MeOH. P1 was set to 232 µL/min and P2 to 468 µL/min. The liquid feeds were combined in a Y-mixer, and the resulting stream was directed through a 3.5-mL reaction coil at 100 °C. The product mixture leaving the coil was collected for 16 min under steady state conditions. The collected mixture was neutralized (pH 7) with a 4 M aq. NaOH solution and was next purified by column chromatography using a 6–16% gradient of MeOH in CH_2Cl_2 as eluent. EIDD-2801 was isolated in 69% yield (307 mg) and ≥99% purity as a white solid.
^{1}H-NMR (300 MHz, MeOH-*d₄*) δ 6.91 (d, *J*= 8.3 Hz, 1H), 5.82 (d, *J*= 4.8 Hz, 1H), 5.61 (d, *J*= 8.2 Hz, 1H), 4.29 (d, *J*= 3.6 Hz, 2H), 4.15-4.07 (m, 3H), 2.62 (sept, *J*= 7.0 Hz, 1H), 1.18 (d, *J*= 7.0 Hz 6H); ^{13}C-NMR (75 MHz, MeOH-d_4) δ 178.2, 151.5, 146.1, 131.7, 99.5, 90.4, 82.5, 74.3, 71.5, 64.9, 35.1, 19.3, 19.3. The NMR data is in agreement with previously published values.[2] HRMS (ESI, positive mode): *m/z* $[M + H]^+$ Calcd for $[C_{13}H_{20}N_3O_7 +H]^+$: 330.1296, found: 330.1297.

8. NMR Spectra

^1H NMRs: 300 MHz, ^{13}C NMRs: 75 MHz

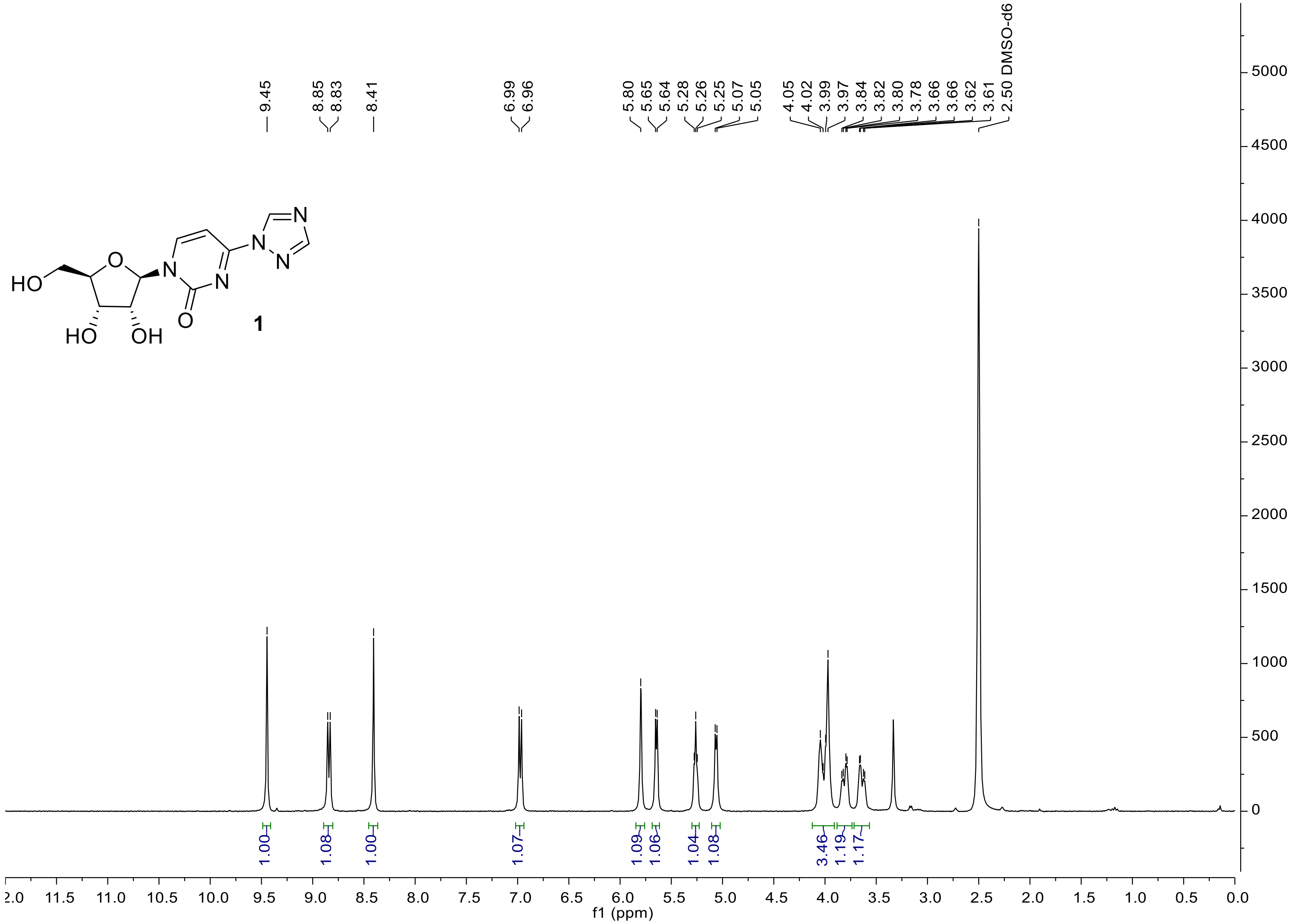

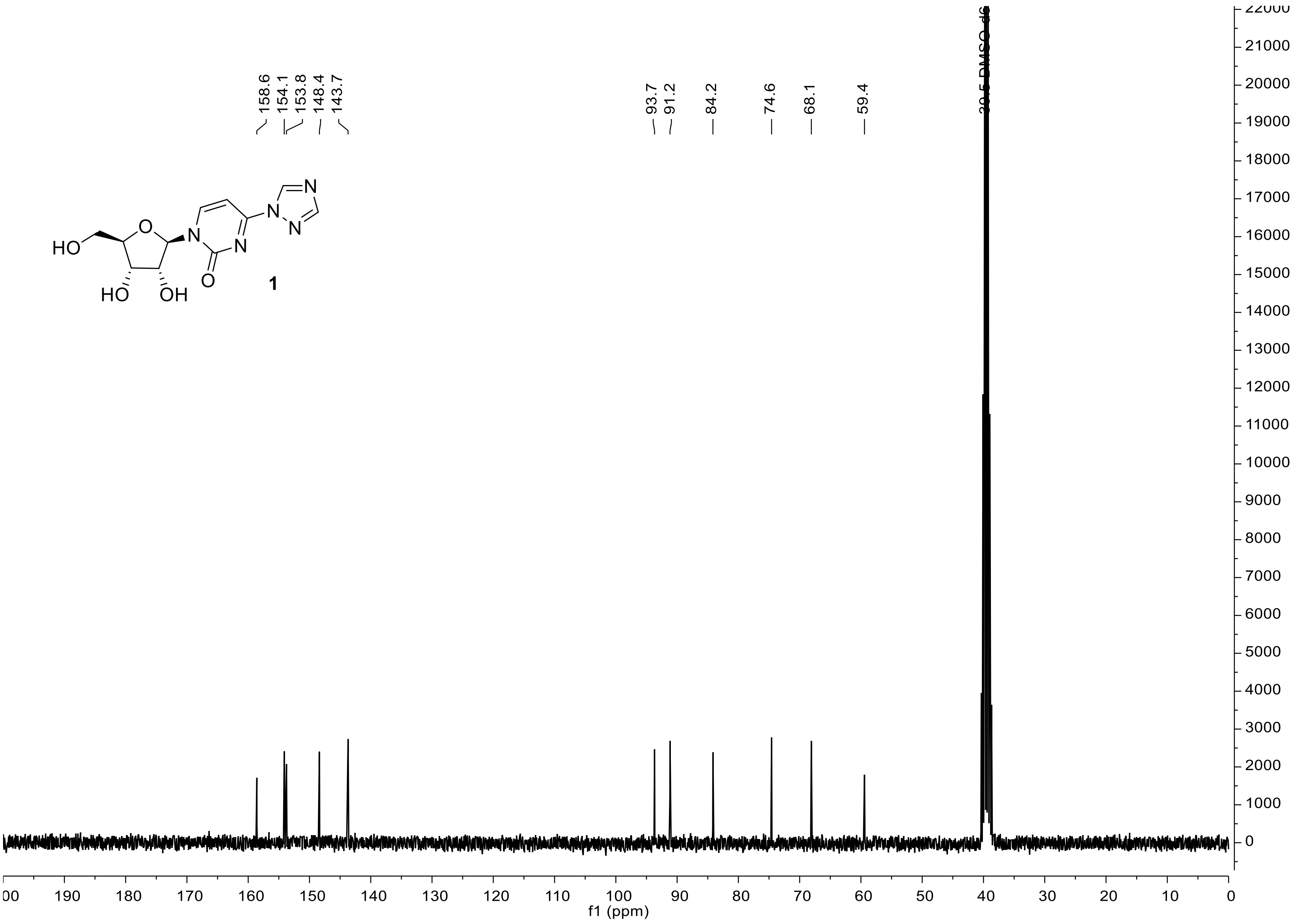
158.6
154.1
153.8
148.4
143.7
93.7
91.2
84.2
74.6
68.1
59.4
HO
O
N
N
O
HO
OH
N
N
N
1
f1 (ppm)
200 190 180 170 160 150 140 130 120 110 100 90 80 70 60 50 40 30 20 10 0
22000 21000 20000 19000 18000 17000 16000 15000 14000 13000 12000 11000 10000 9000 8000 7000 6000 5000 4000 3000 2000 1000 0

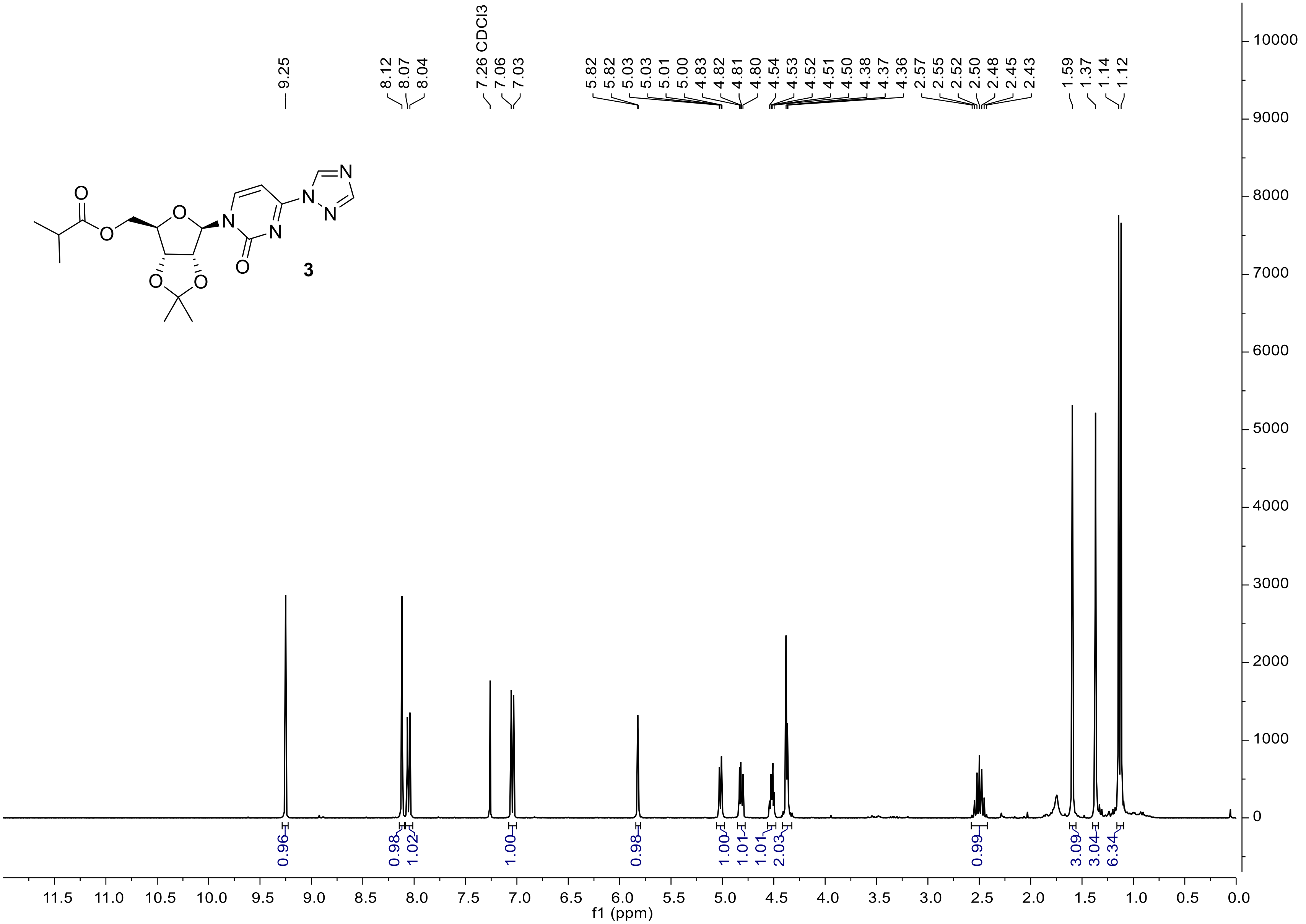
9.25
8.12
8.07
8.04
7.26 CDCl3
7.06
7.03
5.82
5.82
5.03
5.03
5.01
5.00
4.83
4.82
4.81
4.80
4.54
4.53
4.52
4.51
4.50
4.38
4.37
4.36
2.57
2.55
2.52
2.50
2.48
2.45
2.43
1.59
1.37
1.14
1.12
3
0.96
0.98
1.02
1.00
0.98
1.00
1.01
1.01
2.03
0.99
3.09
3.04
6.34
f1 (ppm)

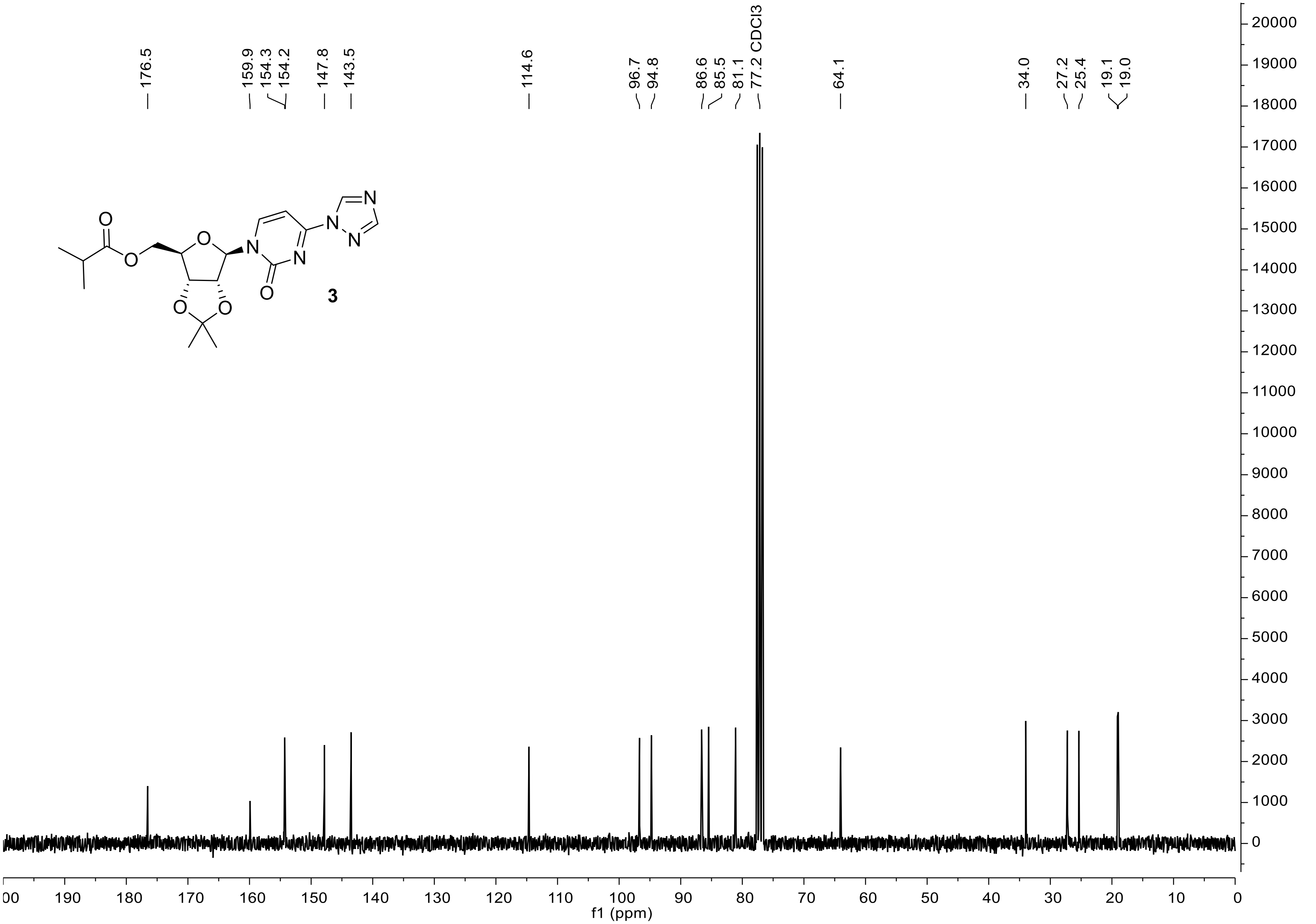
176.5
159.9
154.3
154.2
147.8
143.5
114.6
96.7
94.8
86.6
85.5
81.1
77.2 CDCl3
64.1
34.0
27.2
25.4
19.1
19.0
3
f1 (ppm)

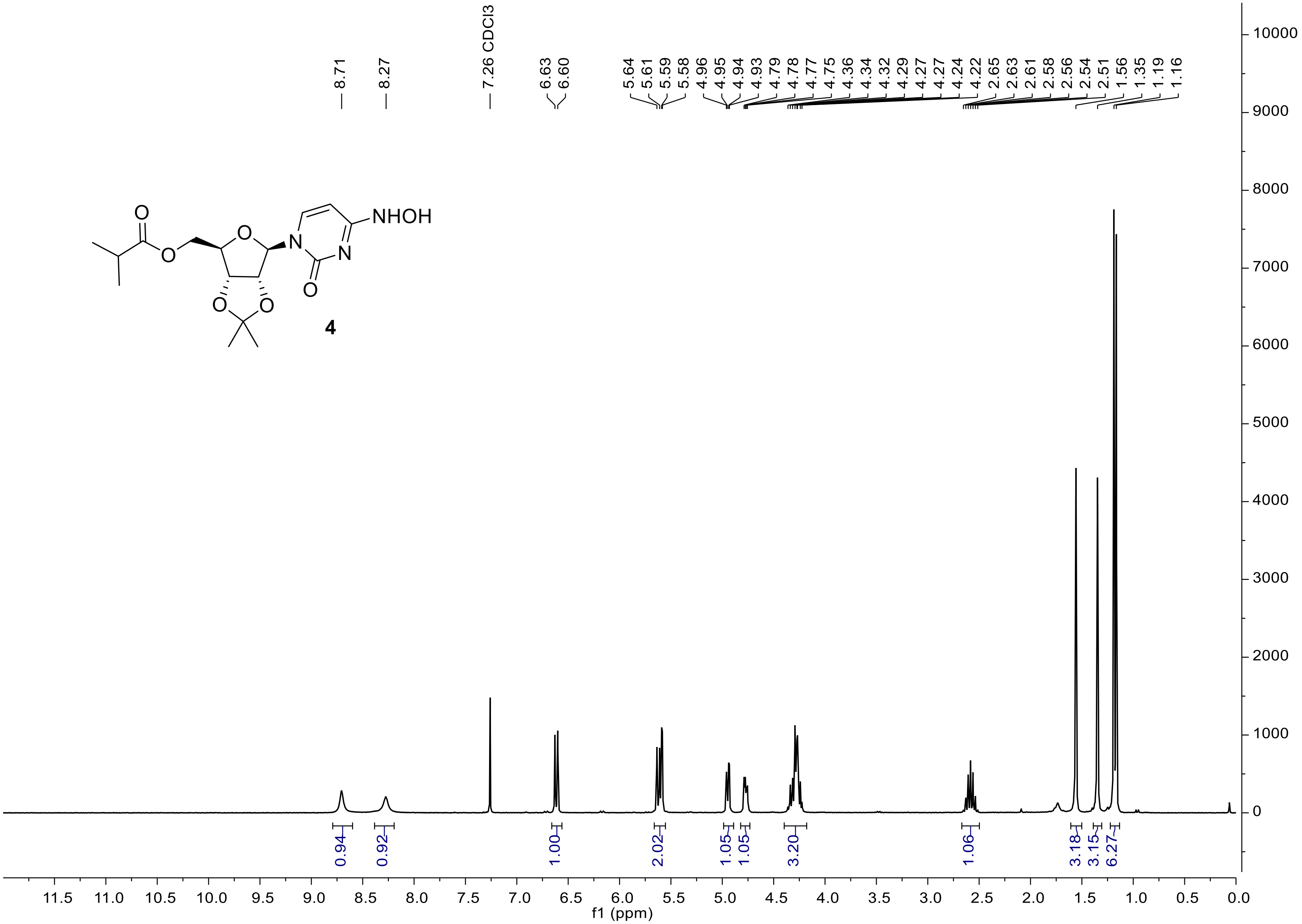
NHOH
4
f1 (ppm)
7.26 CDCl3

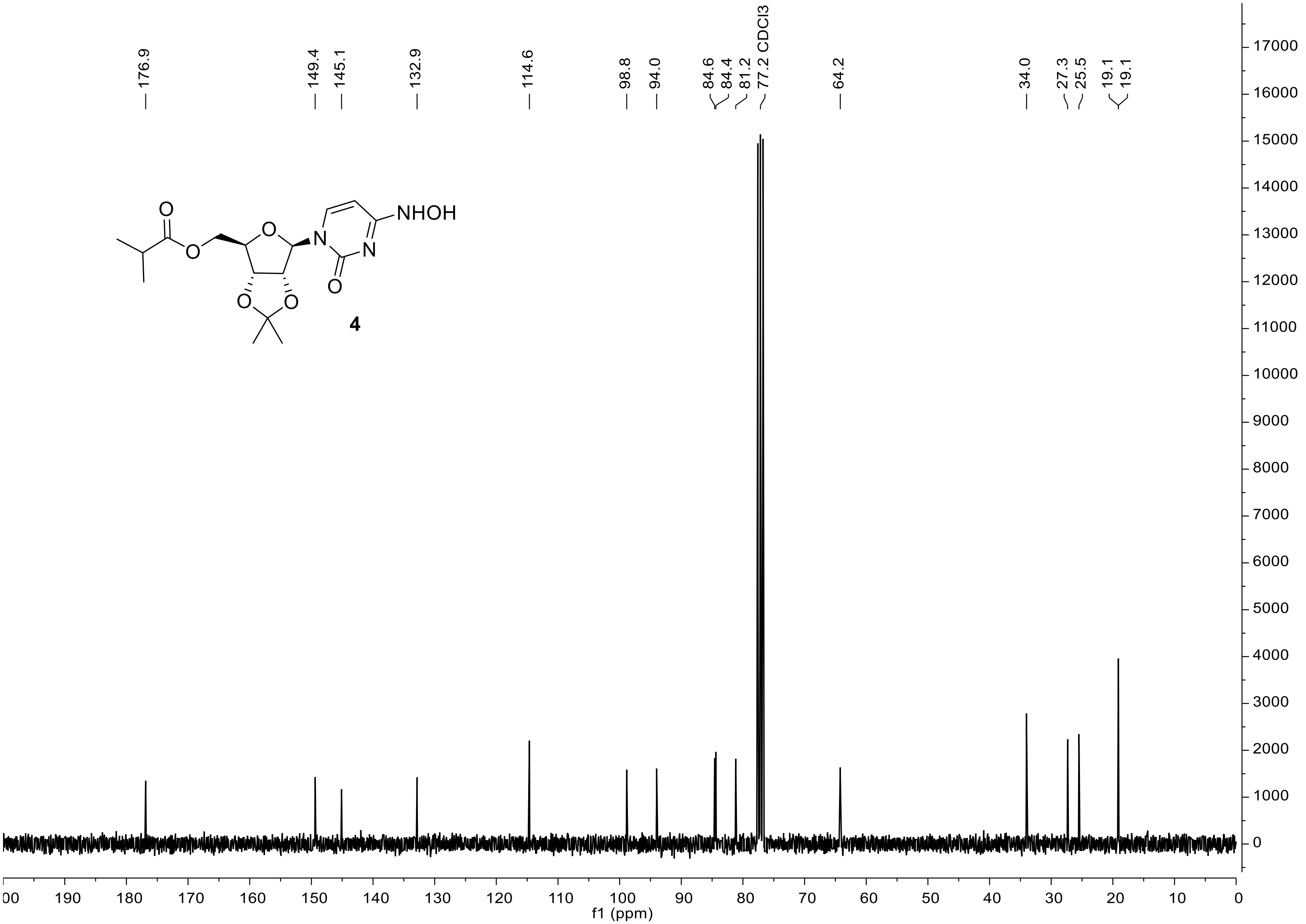
176.9
149.4
145.1
132.9
114.6
98.8
94.0
84.6
84.4
81.2
77.2 CDCl3
64.2
34.0
27.3
25.5
19.1
19.1
NHOH
N
O
4
f1 (ppm)

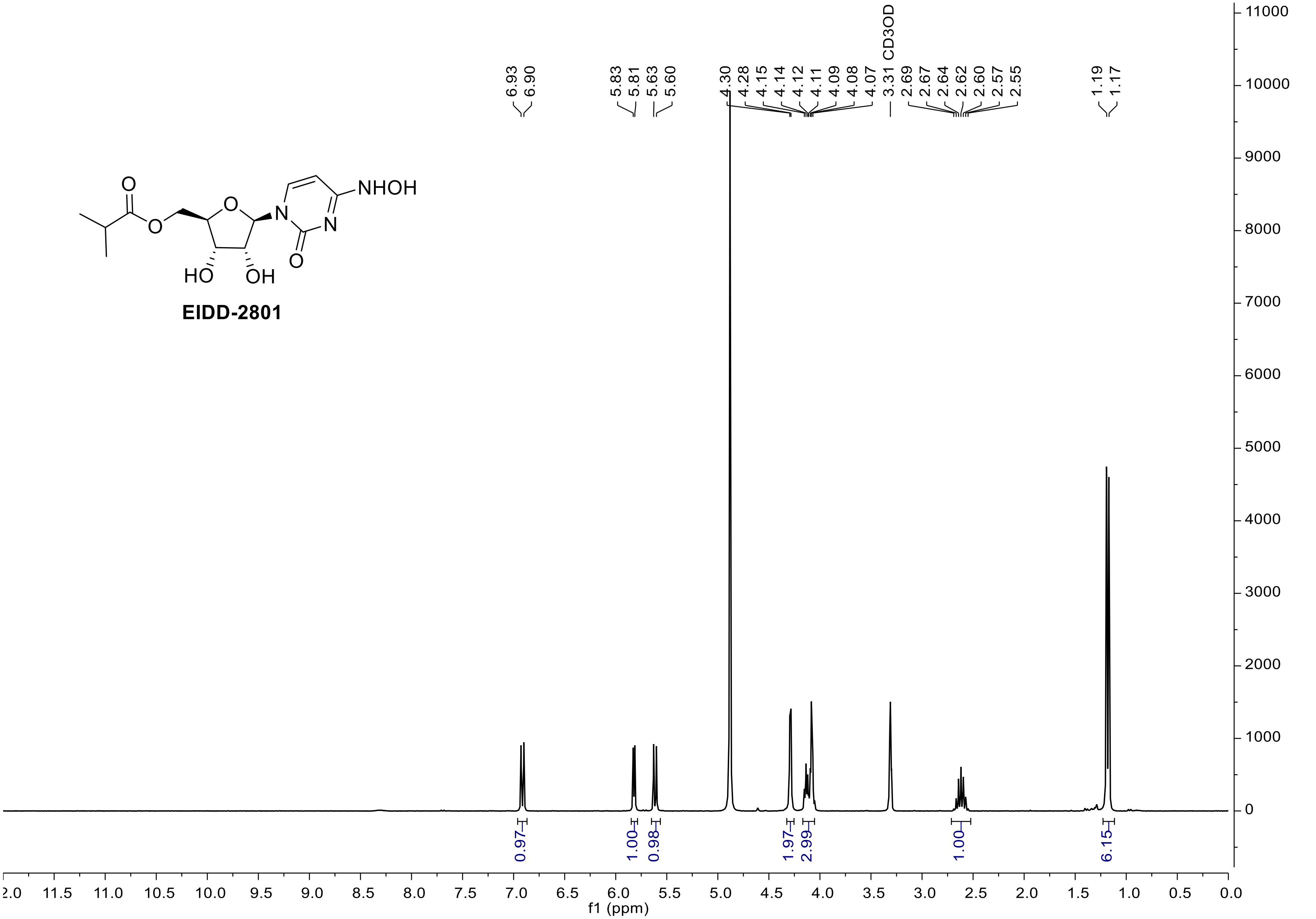

O
O
O
N
NHOH
N
O
HO
OH
EIDD-2801
6.93
6.90
5.83
5.81
5.63
5.60
4.30
4.28
4.15
4.14
4.12
4.11
4.09
4.08
4.07
3.31 CD3OD
2.69
2.67
2.64
2.62
2.60
2.57
2.55
1.19
1.17
0.97
1.00
0.98
1.97
2.99
1.00
6.15
11000
10000
9000
8000
7000
6000
5000
4000
3000
2000
1000
0
2.0
11.5
11.0
10.5
10.0
9.5
9.0
8.5
8.0
7.5
7.0
6.5
6.0
5.5
5.0
4.5
4.0
3.5
3.0
2.5
2.0
1.5
1.0
0.5
0.0
f1 (ppm)

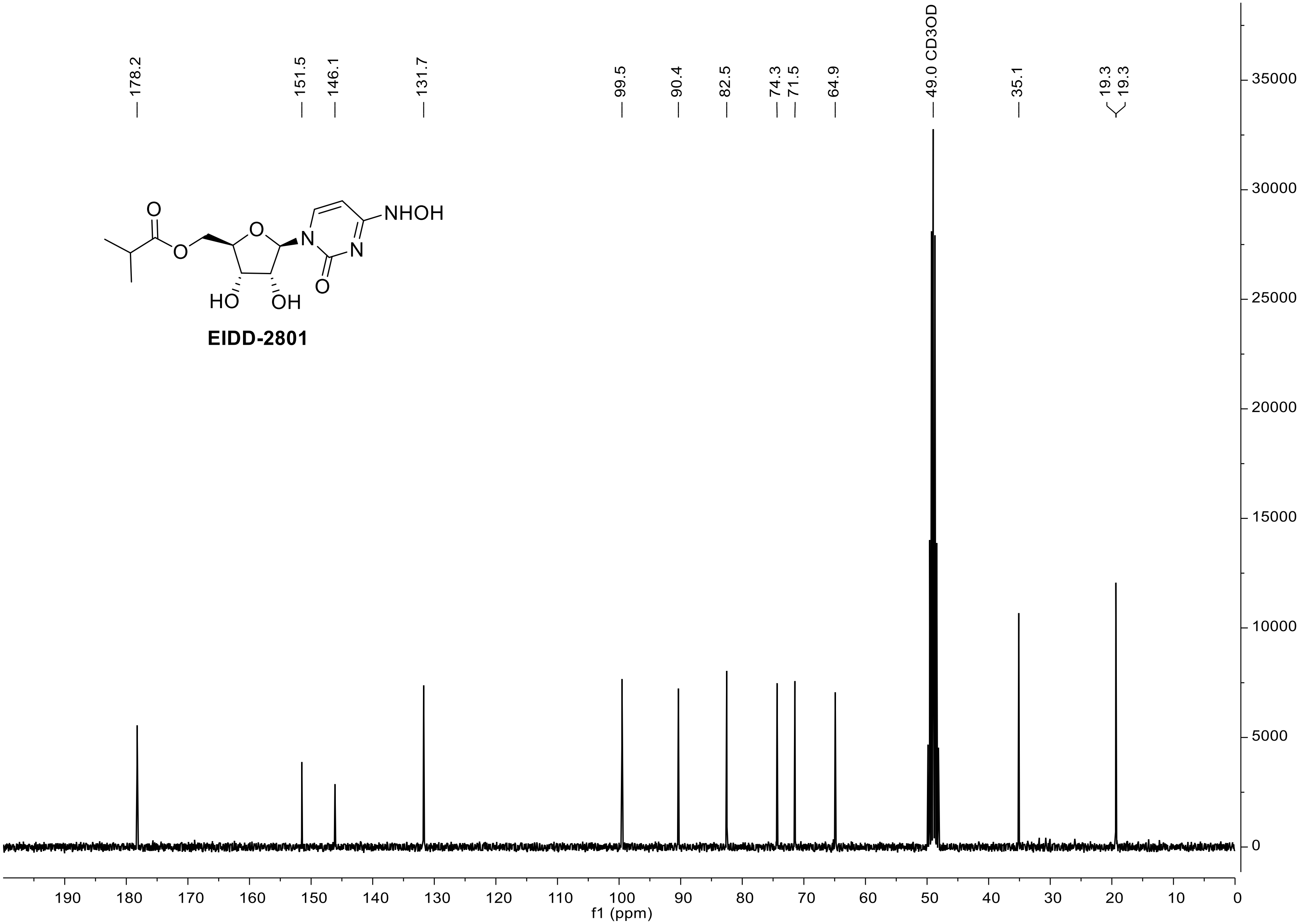

178.2
151.5
146.1
131.7
99.5
90.4
82.5
74.3
71.5
64.9
49.0 CD3OD
35.1
19.3
19.3
NHOH
O
N
HO
OH
EIDD-2801
35000
30000
25000
20000
15000
10000
5000
0
190
180
170
160
150
140
130
120
110
100
90
80
70
60
50
40
30
20
10
0
f1 (ppm)

9. References

[1] A. Miah, C. B. Reese, Q. Song, *Nucleosides and Nucleotides* **1997**, *16*, 53–65.

[2] V. Gopalsamuthiram, C. Williams, J. Noble, T. F. Jamison, B. F. Gupton, D. R. Snead, *Synlett* **2020**, DOI 10.1055/a-1275-2848.
